# Digital health solutions for chronic disease physical activity management: wearable devices, artificial intelligence, and public health implementation

**DOI:** 10.3389/fpubh.2026.1888001

**Published:** 2026-08-04

**Authors:** Linshen Yang, Xiaofeng Wang

**Affiliations:** 1 School of Philosophy and Society, Jilin University, Changchun, China; 2 College of Physical Education, Jilin University, Changchun, China

**Keywords:** artificial intelligence, chronic disease management, digital health, health equity, physical activity, public health implementation, remote rehabilitation, wearable devices

## Abstract

Physical inactivity and sedentary behavior are major modifiable risk factors in chronic disease management, yet conventional clinic-based follow-up is poorly suited to capture real-world behavioral dynamics or deliver timely personalized support. Wearable devices provide longitudinal data on steps, activity intensity, sedentary time, sleep, heart rate, and selected physiological signals, while artificial intelligence (AI) may support feedback personalization, risk prediction, dynamic goal setting, and remote coordination. This review synthesizes evidence on wearable- and AI-supported physical activity management across diabetes, obesity, cardiovascular disease, chronic respiratory disease, cancer survivorship, and older-adult multimorbidity. Current evidence most consistently supports improvements in behavioral outcomes, including steps, physical activity levels, self-monitoring, and in some cases sedentary behavior. Evidence for functional and intermediate clinical outcomes is promising but heterogeneous, while evidence for long-term clinical endpoints, cost-effectiveness, and health-system integration remains less definitive. AI-specific evidence remains comparatively early, heterogeneous, and often feasibility-oriented, so claims about AI-enabled benefit require cautious interpretation. The review argues that wearable devices and AI should not replace clinical care, but should be understood as components of digital public health closed loops that connect continuous sensing, personalized behavioral support, clinical actionability, governance, and equity. Future research should move beyond isolated devices or apps toward validating explainable, actionable, equitable, and sustainable digital intervention pathways in real chronic disease populations and health systems.

## 1 Introduction

Wearable digital health technologies are gradually moving from consumer wellness tools to clinically relevant instruments for longitudinal monitoring, patient engagement, and decision support. Recent discussions have emphasized that their integration into clinical care requires not only accurate sensing, but also careful consideration of data governance, workflow integration, patient trust, and equitable access (1). Long-term chronic disease management is a continuing public health challenge because risk is generated daily, while care is often delivered episodically. Diabetes, obesity, coronary artery disease, heart failure, chronic obstructive pulmonary disease (COPD), cancer survivorship, and multimorbidity in older adults are strongly shaped by everyday behaviors, including insufficient physical activity, prolonged sedentary time, disrupted sleep, and limited self-management capacity. Yet conventional care still relies heavily on clinic visits, episodic measurement, and patient recall. These approaches are limited in their ability to capture real-world behavioral fluctuations, identify declining adherence, or detect early symptom deterioration. Digital health solutions are therefore increasingly discussed not only as patient-facing technologies, but also as tools for extending service reach, supporting continuity of care, and strengthening routine chronic disease management.

Against this background, wearable activity trackers provide a practical means of extending chronic disease monitoring and behavioral support beyond clinical settings. Accumulating evidence suggests that wearable activity trackers can support behavior change by increasing physical activity across both clinical and non-clinical populations. Umbrella-review and meta-analytic evidence further suggests that wearable devices are associated with improvements in physical activity and, in some studies, reductions in sedentary behavior among adults with and without chronic conditions (2, 3). An umbrella review synthesized systematic reviews and meta-analyses and reported that activity trackers can increase physical activity and improve several health-related outcomes (4). Wearable devices have also changed the data entry point for chronic disease management. Consumer activity trackers, smartwatches, continuous heart rate devices, and continuous glucose monitoring systems can record steps, activity intensity, sedentary behavior, sleep, heart rate, rhythm-related signals, and glucose-related indicators, while also estimating energy expenditure over extended periods. Compared with questionnaires, device-measured data provide higher temporal resolution and can capture short, intermittent, and lifestyle-embedded activity patterns. Device-measured physical activity has also been used to examine future health risk, mortality, and major adverse cardiovascular events. Large-scale wearable-device studies have linked objectively measured activity patterns, including vigorous intermittent lifestyle physical activity and incidental physical activity, with mortality and cardiovascular outcomes (5–8). More recent wearable-device evidence has further examined the health equivalence of different physical activity intensities in relation to mortality, cardiometabolic disease, and cancer, reinforcing the value of objective activity monitoring for chronic disease risk assessment (9). In this sense, wearable devices should be treated as more than step-counting tools. They provide a way to describe behavioral and physiological patterns as digital phenotypes. In chronic disease populations, disease-specific and cardiometabolic evidence gives further support to this role. A systematic review and meta-analysis showed that wearable physical activity tracker interventions were associated with improved physical activity among adults with cardiometabolic conditions, particularly when combined with structured feedback, professional support, or other behavior change components (10).

Beyond the basic question of whether wearable monitoring can measure and feedback physical activity, AI introduces further questions about whether feedback, goals, recommendations, and risk signals can be safely adapted to individual states, behavioral histories, contextual cues, and risk profiles. Reinforcement learning, machine-learning user models, just-in-time adaptive interventions, chatbots, and risk prediction models have moved chronic disease physical activity management away from static health education and toward more personalized and context-sensitive support (11–14).

This expanding evidence base still needs careful interpretation. Current evidence is stronger for behavioral outcomes than for long-term clinical endpoints. Short-term trials and single-center studies are more common than health-system implementation studies. Evidence from digitally literate and relatively advantaged populations is more abundant than evidence from vulnerable groups, rural settings, or low-resource care systems. This review therefore addresses three questions: what levels of evidence currently support wearable- and AI-enabled physical activity management as a digital health solution for chronic disease management; what AI adds beyond ordinary wearable monitoring; and what implementation, governance, and equity conditions are needed before these approaches can move from research efficacy to routine service use.

Compared with existing umbrella and systematic reviews that mainly summarize activity effects, health outcomes, or device accuracy (2, 4, 15), this review makes a different contribution by organizing wearable and AI evidence as a digital closed-loop service problem. Its novelty lies in linking evidence maturity, AI actionability, clinical workflow, governance, and equity rather than treating devices, algorithms, or short-term behavior change as isolated endpoints.

## 2 Methods: review approach and evidence-interpretation framework

This article is a problem-oriented narrative review integrating evidence from systematic reviews, meta-analyses, randomized controlled trials, cohort studies, implementation studies, and high-quality narrative or scoping reviews. The review focuses on physical activity, sedentary behavior, exercise prescription, remote rehabilitation, self-management, wearable monitoring, and AI-enabled feedback in adults with chronic diseases or chronic disease risk. To avoid reducing wearables to step-counting tools, this review also includes evidence on chronic disease self-management and remote monitoring to clarify the role of wearables in symptom recording, continuous physiological monitoring, patient feedback, and care coordination (16, 17).

The reporting and appraisal of this narrative review were guided by the SANRA principles for narrative review articles, including justification of the article's importance, statement of aims, literature-search description, appropriate referencing, scientific reasoning, and balanced presentation (18). The review remains narrative rather than systematic; the search log was used to improve transparency, not to claim PRISMA-style exhaustive evidence capture.

The review used a qualitative narrative synthesis with thematic evidence mapping. It did not generate primary quantitative data, statistically pool effect sizes, or claim to be a systematic review. Instead, the aim was to integrate high-level and disease-specific evidence to clarify how wearable devices and AI may support chronic disease physical activity management, and under what implementation, governance, and equity conditions such support is likely to be clinically and publicly meaningful.

The literature search covered publications from January 2015 to May 2026. The main searches were conducted in Web of Science Core Collection and Google Scholar, supplemented by backward citation tracking and final citation-status verification before the original submission. Detailed search themes, search strings, topic-specific search dates, and source-selection counts are provided in Supplementary Table S1. In brief, 356 records/items were identified, 112 full texts were assessed, and 71 core sources were retained for detailed narrative synthesis. Because Google Scholar yields vary over time, relevance-ranked results were screened within the predefined thematic scope.

The final 71 sources were selected according to four criteria: relevance to adult chronic disease or chronic disease risk; direct relevance to wearable monitoring, AI-enabled feedback/prediction, mHealth-supported physical activity management, or remote rehabilitation; contribution to at least one evidence function (effectiveness, risk measurement, implementation, or governance/equity); and ability to support the cross-disease evidence hierarchy used in this review. References dated 2026 were included only when final journal publication information was available within the May 2026 search and verification window. Quantitative values were rechecked against the cited sources during revision; uncertainty intervals were retained when reported in the source and relevant to the narrative argument.

Eligible sources addressed adults with chronic diseases or chronic disease risk and reported evidence relevant to wearable monitoring, AI-enabled feedback or prediction, mHealth-supported physical activity management, remote rehabilitation, chronic disease self-management, or public health implementation. Systematic reviews, meta-analyses, randomized trials, large cohort studies, implementation studies, feasibility or acceptability studies, scoping reviews, narrative reviews, and professional statements were considered. Sources were excluded when they focused only on athletic performance, children or adolescents, acute-care monitoring without physical activity or self-management relevance, purely technical sensor development without health-management implications, short commentaries without substantive evidence synthesis, non-English texts, or records without accessible full text.

Evidence was identified and organized iteratively. First, sources were grouped by disease context and technology function. Second, the main evidence contribution of each source was coded as effectiveness evidence, risk-measurement evidence, implementation evidence, or conceptual/governance evidence. Third, findings were mapped to the outcome hierarchy described below. This approach was used to avoid treating all wearable or AI studies as equivalent and to distinguish short-term behavioral effects from functional, clinical, service-level, and equity-related implications.

Evidence is interpreted through a three-level framework. The first level is behavioral outcomes, including steps, moderate-to-vigorous physical activity (MVPA), sedentary time, wearing rate, adherence, and self-management behavior. The second level is functional and intermediate clinical outcomes, including cardiorespiratory fitness, exercise tolerance, HbA1c, glucose variability, body weight, physical function, symptom burden, and quality of life. The third level is hard endpoints, including major adverse cardiovascular events (MACE), hospitalization, mortality, atrial fibrillation, chronic disease incidence, and long-term complications. This hierarchy prevents short-term behavior change from being overinterpreted as long-term clinical benefit.

To further improve interpretive transparency, three evidence functions are distinguished. The first is effectiveness evidence, mainly from randomized trials, systematic reviews, and meta-analyses, which evaluates whether wearable or digital interventions improve physical activity, sedentary behavior, functional outcomes, or metabolic indicators. The second is risk-measurement evidence, mainly from device-measured cohorts, which evaluates whether steps, activity intensity, sleep, and sedentary patterns are associated with chronic disease risk or hard endpoints. The third is implementation evidence, including qualitative studies, feasibility studies, acceptability surveys, scientific statements, and evaluation frameworks, which assesses whether technologies can be sustainably used by real patients, primary care services, and clinical teams. These evidence types answer different questions and should not be substituted for one another.

Four principles guide the synthesis. First, systematic reviews, meta-analyses, large-scale cohorts, and professional statements are prioritized as core evidence, while individual trials and implementation studies are used to explain mechanisms and translation conditions. Second, disease contexts are not forced into a single conclusion; instead, each context is discussed through the chain of disease burden, behavioral target, digital function, evidence strength, and implementation barrier. Third, consumer wearables, medical-grade monitoring devices, mHealth applications, and AI algorithms are conceptually distinguished rather than merged into a single digital intervention category. Fourth, equity, data quality, and workflow integration are treated as components of effectiveness rather than peripheral issues after technological maturity.

**Figure 1:**
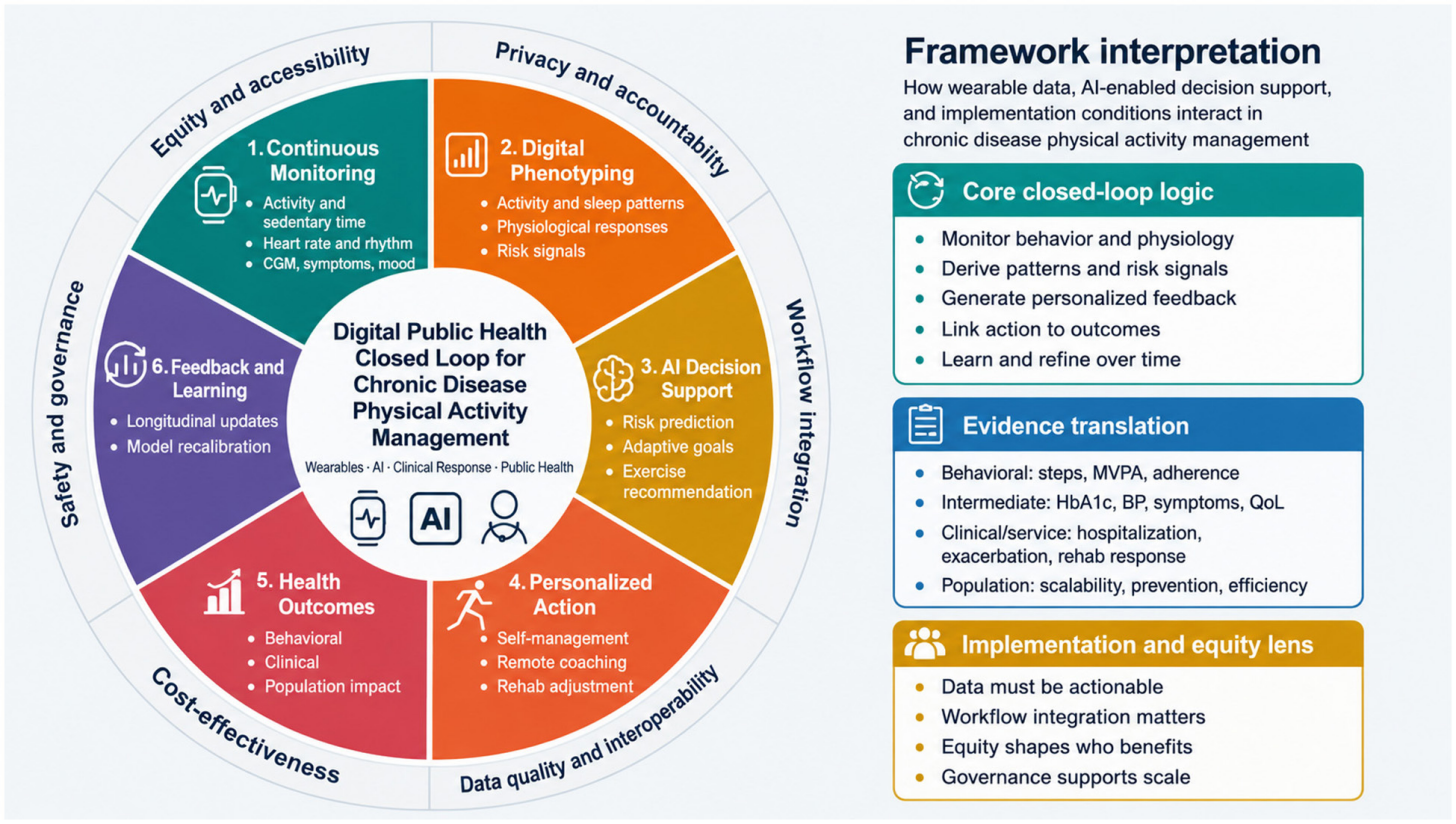
Wearable- and AI-enabled digital public health closed loop for chronic disease physical activity management. Continuous monitoring generates behavioral, physiological, metabolic, and patient-reported data that can be translated into digital phenotypes and risk signals. AI-enabled decision support provides personalized goals, adaptive feedback, and exercise-related recommendations, informing patient self-management, remote coaching, clinical review, and rehabilitation adjustment. These actions lead to behavioral, functional, clinical, service, and population-level outcomes, which feedback into longitudinal learning and service refinement. Implementation, governance, and equity conditions are essential for sustainable and equitable digital public health practice.

Theoretically, wearable- and AI-enabled physical activity management involves at least three mechanism chains. The first is a self-regulation chain, in which continuous monitoring improves behavioral visibility, feedback and goal setting promote self-comparison, and activity execution becomes more sustained. The second is a clinical coordination chain, in which remote data allow clinicians to detect risk earlier, adjust prescriptions, and arrange follow-up. The third is an algorithmic adaptation chain, in which AI modifies intervention timing, content, and intensity based on prior behavior, context, and response. A digital intervention can move from short-term behavior change to longer-term health benefit only when data capture, behavioral feedback, and clinical response operate as an integrated loop. This conceptual framework is summarized in Figure 1, and the evidence maturity structure is summarized in Table 1.

**Table 1:** Current evidence maturity levels for wearable- and AI-enabled chronic disease physical activity management.

Evidence level	Main outcomes	Current maturity	Supported interpretation
Behavioral level	Steps, moderate-to-vigorous physical activity, sedentary behavior, adherence, self-monitoring	Relatively high	Wearable devices and digital feedback can improve physical activity behavior.
Functional/intermediate level	HbA1c, blood pressure, body weight, cardiorespiratory fitness, exercise tolerance, quality of life	Moderate	Improvements are observed in some populations and multicomponent interventions, but heterogeneity is substantial.
Hard endpoint level	Major adverse cardiovascular events, mortality, hospitalization, atrial fibrillation, exacerbation, chronic disease incidence	Emerging	Device-measured activity is associated with risk; intervention-induced reductions in hard endpoints require stronger causal evidence.
Implementation level	Wearing rate, acceptability, workflow, cost, equity, governance	Insufficient	Implementation and equity conditions are major constraints in translation from research to routine service.

## 3 Overall evidence on wearable devices: behavioral improvement is strongest, endpoint evidence is still accumulating

Cross-disease reviews and umbrella reviews show relatively consistent evidence that wearable devices can increase physical activity. One umbrella review including 51 systematic reviews, 38 of which contained meta-analyses and 302 independent primary studies, reported that wearable device use was associated with increased steps and MVPA. The median effect was approximately 1,312 additional steps per day and 57.8 additional minutes of MVPA per week, although sedentary behavior outcomes were inconsistent and 72.5% of included reviews were rated as having critically low methodological quality (2). A meta-analysis of consumer activity tracker interventions in chronic disease populations included 35 studies across chronic respiratory disease, type 2 diabetes, cardiovascular disease, overweight/obesity, cognitive impairment, and sedentary older adults. It found an average increase of approximately 2,123 steps per day, accompanied by improvements in systolic blood pressure, waist circumference, and LDL cholesterol (3). These findings suggest that the most direct and defensible function of wearable devices is behavioral visualization and feedback reinforcement, not automatic improvement in clinical endpoints.

Wearable devices influence behavior through three linked mechanisms. First, they convert otherwise invisible daily activity into trackable indicators, enhancing self-monitoring. Second, goal setting, reminders, rewards, and social comparison make behavior change techniques concrete. Third, continuous data allow clinical teams or digital coaches to identify declining adherence and behavioral fluctuations. Studies suggest that wearable use combined with feedback, coaching, or behavioral support is more likely to produce sustained change than passive device wearing alone (19–21).

Endpoint evidence comes primarily from large-scale device-measured cohorts. UK Biobank and All of Us studies suggest that device-measured physical activity, sleep, and everyday activity patterns are associated with chronic disease risk, MACE, and mortality (22, 23). These findings increase the public health significance of wearable-derived indicators, but they remain largely observational. They support the claim that device-measured behavior has risk-stratification value, but not the stronger claim that providing devices necessarily reduces event risk.

The meaning of effectiveness in wearable research is therefore multi-layered. For individual patients, increased steps, reduced sitting, and improved self-efficacy may already be meaningful. For clinicians, the critical question is whether digital indicators support prescription adjustment, risk stratification, and treatment decisions. For health systems, the important question is whether digital interventions can reach high-risk populations at acceptable cost and reduce avoidable hospitalizations or functional decline. Reviews in noncommunicable disease management also show that wearables frequently appear within broader lifestyle-management systems involving physical activity and nutrition, making it insufficient to attribute all effects to the device alone (24). Real-world survey evidence further suggests that wearable use among adults with chronic conditions is shaped by age, education, health status, and technology access; trial adherence should not be directly generalized to the broader chronic disease population (25).

Wearable measurement also changes physical activity science itself. Traditional questionnaire-based research poorly captures short bouts, intensity fluctuations, sedentary interruptions, and all-day movement patterns. Wearables make micro-doses of activity, incidental activity, vigorous intermittent lifestyle activity, and sleep–activity combinations quantifiable. This creates the possibility of intervention targets beyond weekly exercise duration, including daily activity fragments, sedentary breaks, sleep rhythms, and risk-specific behavioral windows. This shift from structured exercise to an all-day behavioral spectrum is one of the main theoretical contributions of wearables to chronic disease management.

**Figure 2:**
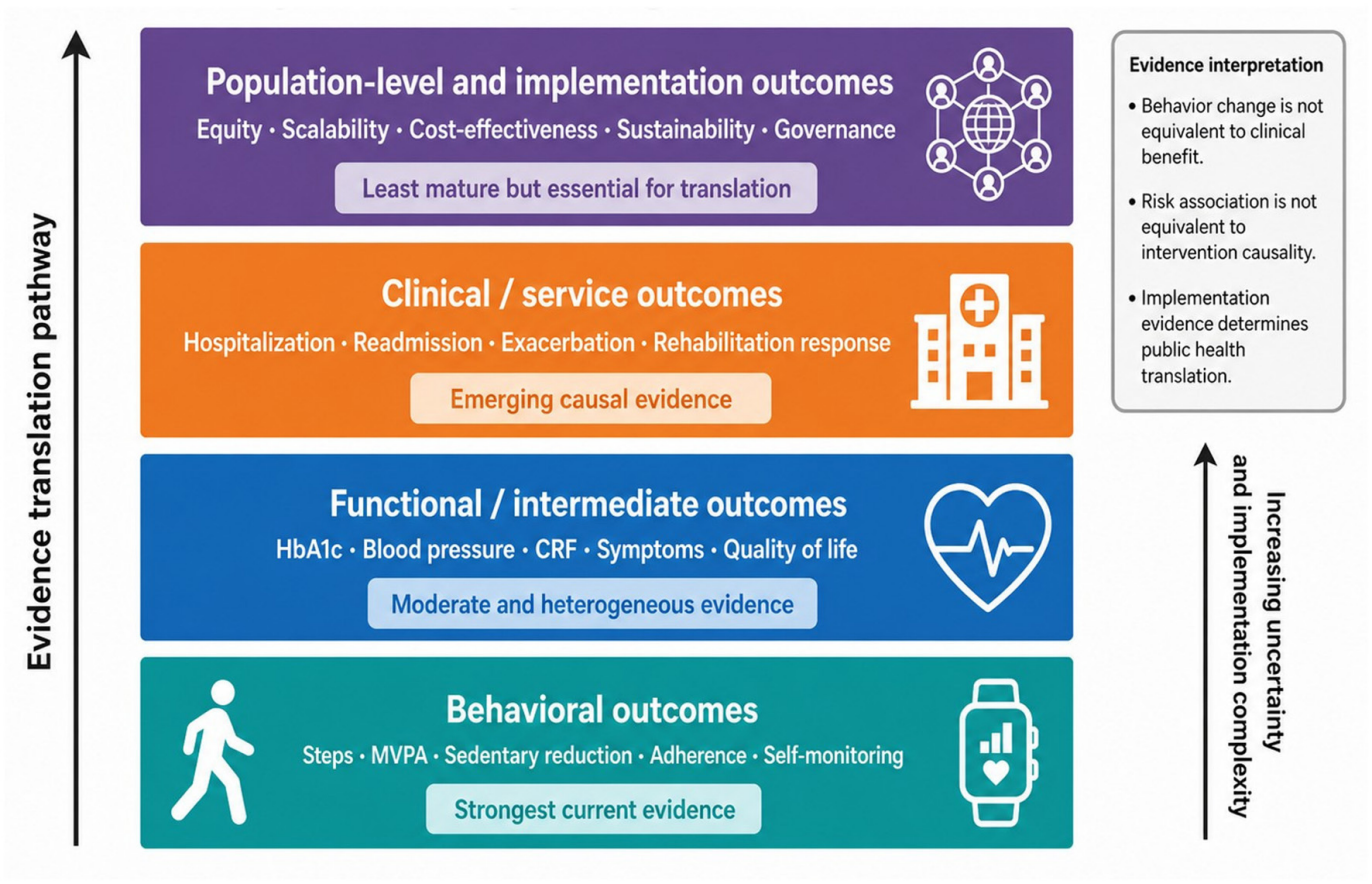
Levels of evidence for wearable- and AI-enabled physical activity management in chronic disease. The hierarchy distinguishes behavioral outcomes, functional and intermediate outcomes, clinical and service outcomes, and population-level or implementation outcomes. It also indicates that current evidence is strongest for behavioral changes and more limited for hard endpoints and system-level outcomes.

At the same time, higher frequency does not automatically imply higher quality. Device sensors, wearing positions, algorithm versions, firmware updates, and cleaning procedures influence estimates of steps, energy expenditure, sleep, and heart rate. High-level reviews should avoid treating wearable outputs as objective gold standards. They are algorithmically processed digital phenotypes whose clinical value depends on accuracy, repeatability, interpretable thresholds, and actionable response rules. Figure 2 presents the proposed hierarchy of evidence levels.

## 4 Evidence across chronic disease contexts

### 4.1 Metabolic diseases: from step feedback to glucose–behavior loops

Diabetes and obesity are among the most evidence-rich contexts for wearable-enabled physical activity management. Adults with type 2 diabetes commonly experience insufficient activity, prolonged sitting, glucose variability, and high self-management burden. A 2024 systematic review and meta-regression of 19 randomized controlled trials in adults with type 2 diabetes found that wearable technology-based physical activity interventions increased steps by approximately 1,583 per day and reduced systolic blood pressure by approximately 2.46 mmHg. Subgroup and meta-regression analyses suggested that intervention function, duration, age, and goal-setting components may influence effects (26). Another meta-analysis of 25 randomized trials found that wearable activity monitor interventions were associated with small improvements in HbA1c, BMI, waist circumference, and daily steps, although step outcomes were heterogeneous and long-term evidence remained insufficient (27). Thus, in type 2 diabetes, wearable interventions can be stated relatively confidently to improve physical activity behavior, while claims regarding HbA1c, body weight, and long-term complications should be made cautiously.

Several factors explain inconsistency in metabolic outcomes. Glucose control and body weight are multifactorial outcomes influenced by diet, medication, sleep, disease duration, stress, and social environment. A single activity tracker is unlikely to produce strong metabolic effects. Intervention intensity also varies widely, ranging from self-tracking alone to multicomponent programs including coaching, text messages, apps, continuous glucose monitoring (CGM), and clinical feedback. Declining adherence weakens both behavior change and data completeness. Evidence from type 2 diabetes self-tracking, primary-care activity tracker implementation, and sedentary-behavior process evaluations indicates that patient acceptability, clinical workflow alignment, understandable goals, and actionable feedback determine whether the technology can be implemented (28–30).

An important shift in diabetes is the movement from activity tracking toward glucose–behavior feedback. CGM is not only a monitoring device but also a behavior change tool, because it helps individuals understand the relationships among diet, activity, sleep, and glucose (31). Studies integrating digital trackers with CGM further suggest that combining exercise, diet, and glucose data supports more refined self-management (32). This provides a stronger mechanistic basis for closed-loop management of physical activity and metabolism.

AI evidence in diabetes is relatively concrete. Early reinforcement learning studies suggested that systems can adjust feedback according to individual responses, thereby promoting physical activity and glycemic management (33, 34). The DIAMANTE randomized clinical trial provides stronger evidence. It recruited 168 patients with diabetes and depressive symptoms from four San Francisco public primary care clinics and online platforms; 24% preferred Spanish and 37.5% were recruited through clinics. Participants received weekly mood monitoring, random messages, or reinforcement learning-based adaptive messages. At 24 weeks, the adaptive arm showed a sustained increase in daily steps, with an estimated total increase of approximately 608 steps and an approximately 19% increase after intervention, compared with smaller increases in random message and control groups (14). Diabetes is therefore a useful context for demonstrating how AI can transform fixed reminders into response-adaptive behavioral support.

For metabolic diseases, the critical issue is not simply making patients walk more, but building interpretable behavior–metabolism feedback. Many patients struggle to understand how postprandial walking, sedentary breaks, sleep, stress, and medication jointly influence glucose variability. Integration of CGM, activity tracking, and dietary records allows digital systems to move from generic advice toward contextual recommendations, such as identifying postprandial hyperglycemia windows, prompting low-intensity activity breaks, or adapting daily goals based on prior sleep and activity load.

Metabolic digital interventions are nevertheless vulnerable to overinterpretation. HbA1c requires sufficient duration and intensity of behavior change; short-term step increases may not translate into significant glycemic improvements. Weight change also depends on energy intake, medication, and social context. Thus, a tiered interpretation is appropriate: steps, physical activity, and self-monitoring can be discussed confidently; HbA1c, weight, and glucose variability should be discussed as cautiously positive; long-term complications and health service use remain insufficiently supported. The most valuable future designs will integrate wearable activity data, CGM, medication adjustment, and dietary behavior into the same trial framework and prespecify mediating pathways from sedentary breaks or postprandial activity to glucose variability and HbA1c.

### 4.2 Cardiovascular diseases: from remote rehabilitation to hard endpoint risk stratification

Cardiovascular evidence follows two main lines. One concerns wearable and digital support for rehabilitation and secondary prevention. The other concerns device-measured physical activity as a risk phenotype associated with cardiovascular endpoints.

In rehabilitation and secondary prevention, wearables can monitor exercise prescriptions, support home-based rehabilitation, promote adherence, and provide remote feedback. A meta-analysis of wearable-based cardiac rehabilitation interventions in secondary prevention of coronary artery disease searched up to March 2025 and included 23 randomized trials, 20 of which were included in quantitative synthesis. The total sample was approximately 2,964, with a mean age of 61.5 years, 20% women, and a mean intervention duration of 25 weeks. Wearable-supported rehabilitation increased daily steps by approximately 1,060 and reduced rehospitalization risk, while also producing small improvements in six-minute walk distance and absolute peak oxygen uptake (35). A systematic review of smartphone applications for physical activity and sedentary behavior in cardiovascular disease included 19 studies and 1,543 participants across coronary heart disease, hypertension, stroke, heart failure, and peripheral arterial disease. Meta-analysis of 13 studies suggested increases in MVPA of approximately 40.35 min per week and daily steps of approximately 2,390, although risk of bias was high and sedentary outcomes were insufficiently reported (36). A randomized trial of sedentary behavior reduction also suggests that reducing sitting may influence cardiovascular health, skeletal muscle oxidative capacity, and functional exercise capacity, supporting sedentary interruption as an independent target (37).

Device-measured cohort studies have transformed cardiovascular physical activity epidemiology. A prospective cohort study using wrist-worn accelerometer data from UK Biobank analyzed 25,241 adults who did not engage in leisure-time exercise. The mean age was 61.8 years and mean follow-up was 7.9 years, during which 824 MACE and 1,111 deaths occurred. Compared with vigorous intermittent activity bouts lasting less than one minute, longer bouts of one to under 3 min, three to under 5 min, and five to under 10 min were associated with lower all-cause mortality and similar reductions in MACE risk (7). A cohort study of incidental physical activity among non-exercising adults found L-shaped dose-response associations with MACE, cardiovascular mortality, and all-cause mortality, with risk reductions even at small daily volumes of incidental vigorous activity (8). Among 49,060 UK Biobank participants with hypertension, device-measured moderate-intensity activity showed inverse dose-response associations with all-cause mortality and cardiovascular outcomes, while benefits of very high-intensity activity were not necessarily linear (38).

These findings have two implications. First, wearables are not only intervention tools but also exposure-measurement tools that provide more granular evidence for the public health value of small amounts of activity. Second, observational hard-endpoint evidence cannot replace intervention evidence. It is more appropriate to conclude that device-measured activity patterns have risk-stratification and target-identification value than to claim that wearables have already been proven to reduce MACE.

AI-enhanced cardiovascular monitoring deserves separate attention. The SAFEHEART multicenter prospective study of AI-enhanced wearable technology for ventricular arrhythmia prediction recruited 303 implantable cardioverter-defibrillator patients who wore accelerometers for 365 days. The final analysis included 277 patients, 56 ventricular arrhythmia events, and 64,995 days of behavioral data. Twenty-eight-day behavior time series were transformed into statistical summaries and neural network deep representations; reduced daily activity segments and lower day-to-day variability in sleep features were associated with subsequent arrhythmia risk. The deep representation achieved an area under the receiver operating characteristic curve (AUROC) of approximately 0.74, outperforming statistical summaries at approximately 0.67 (39). This indicates that AI can extract dynamic behavioral risk signals that conventional summary features may miss.

Cardiovascular disease also illustrates the centrality of safety. Physical activity is therapeutic but may be risky in high-risk patients. Wearables can support activity promotion, heart rate response monitoring, abnormal rhythm detection, stratified exercise prescription, and safety boundaries for home rehabilitation. Evidence linking physical activity and cardiorespiratory fitness to adverse outcomes in atrial fibrillation suggests that device-measured activity and function may supplement risk stratification in rhythm-disorder populations (40). Reviews and surveys in heart failure further suggest interest in remote support and self-care tools, but uptake depends on symptoms, willingness, and nursing workflow (41, 42).

From a health service perspective, cardiac rehabilitation is one of the most promising use cases for wearable implementation. Center-based rehabilitation is limited by distance, time, cost, and accessibility. Home-based or hybrid rehabilitation supported by wearables may expand coverage, but only if embedded in rehabilitation workflows. Who reviews data, who responds to abnormalities, how prescriptions are adjusted, how records enter electronic health systems, and when in-person evaluation is required must be defined. Without these arrangements, remote rehabilitation may become unsupported app use rather than a clinical service.

### 4.3 COPD and pulmonary rehabilitation: monitoring is valuable, AI closed loops remain underdeveloped

Physical activity decline in COPD is associated with symptom burden, exacerbation risk, and quality of life. Pulmonary rehabilitation is effective, but center-based or hospital-based programs face accessibility and continuity problems. Wearables provide tools for home-based rehabilitation and daily activity monitoring. A 2026 network meta-analysis of COPD digital health interventions included 22 randomized trials and 1,556 participants, comparing synchronous telerehabilitation, multicomponent digital interventions, wearable-only interventions, center-based pulmonary rehabilitation, and usual care. Synchronous telerehabilitation ranked highest for daily steps, while wearable-only interventions ranked highest for six-minute walk distance, but certainty was low and network inconsistency was present (43). Another meta-analysis of wearable devices in chronic respiratory disease rehabilitation included nine studies and 743 patients with COPD or asthma. It found a significant increase in daily steps but inconsistent effects on quality of life, functional capacity, and anxiety (44). A classic COPD pedometer randomized trial involving 102 participants found that the pedometer group increased by approximately 3,080 steps per day, compared with approximately 138 steps in the control group, and improved quality of life and 6-min walk distance (45).

COPD poses specific methodological challenges. Symptom fluctuations, exacerbations, oxygen therapy, cardiovascular comorbidities, and limited exercise tolerance make simple step targets insufficient. Home data are affected by wearing behavior, signal quality, and environmental variability. Wearable monitoring during inpatient pulmonary rehabilitation, reviews on wearable monitoring in COPD, and remote patient monitoring strategies all suggest that devices can record activity and support remote care, but translation into individualized prescriptions requires standardized data processing and clinical feedback protocols (46–48).

The weakest aspect of COPD evidence is AI-enabled closed-loop management. Existing studies emphasize monitoring and remote follow-up rather than integrating wearable data, symptoms, environmental exposure, and individual exercise tolerance into dynamic prescriptions. COPD should therefore be described as a field where evidence for digital pulmonary rehabilitation and remote monitoring is emerging, while AI-personalized closed loops remain early.

COPD also reveals the limitation of single-metric management. Activity reduction is both a result of disease severity and a driver of further functional decline. Dyspnea leads patients to avoid activity, and avoidance worsens deconditioning and fear of exertion. Wearables can transform the subjective sense of low activity into a daily activity profile, enabling rehabilitation teams to examine whether activity decline is linked to exacerbation, weather, oxygen saturation, sleep, or psychological factors. Future integration of symptom diaries, inhaler use, environmental exposure, and activity data may support more individualized warning systems and rehabilitation prescriptions. However, digital pulmonary rehabilitation must report not only steps, but also dyspnea, fatigue, recovery time, symptom scores, quality of life, exacerbations, hospitalizations, and adverse events. Otherwise, the evidence will remain at the feasibility level.

### 4.4 Cancer survivorship and older adults: feasibility and equity are equally important

Cancer survivors and older adults share a key feature: physical inactivity is common, but symptom burden, fatigue, functional decline, and social support vary substantially. A meta-analysis of wearable trackers and pedometers in cancer survivors included 35 randomized trials. Breast cancer studies accounted for 43%; intervention duration ranged from 4 weeks to 1 year; 71% used pedometers and 20% used Fitbit devices. Compared with usual care, wearable or pedometer interventions showed moderate to large effects on moderate-intensity activity, MVPA, total physical activity, and daily steps, and were associated with improved quality of life, aerobic fitness, physical function, and fatigue (49). A meta-analysis of wearable electronic device-supported physical activity programs provides additional randomized trial evidence (50). Trials among regional and remote cancer survivors and home-based mHealth interventions replacing sedentary time in older cancer survivors indicate that remote coaching and home-based intervention may improve accessibility (51, 52).

Older adult evidence also supports behavioral improvement but highlights implementation complexity. A meta-analysis of 23 randomized trials involving 4,566 community-dwelling older adults found that wearable activity tracker interventions increased physical activity time and daily steps but did not consistently improve BMI, body fat, timed-up-and-go performance, or chair-stand tests (53). Another systematic review and meta-analysis of 45 studies and 7,144 participants found increases in daily steps, weekly MVPA, and total daily activity, with small reductions in sedentary time. Effects on MVPA were stronger when trackers were combined with telephone counseling, goal setting, and self-monitoring, and short-term interventions appeared more effective than longer-term interventions (54). A pilot cluster randomized trial in frail older adults further shows that feasibility must be validated separately in vulnerable groups (55).

Equity is central in these populations. Studies on the digital divide among older adults with chronic diseases, ICT-supported chronic disease self-management, and rural digital interventions indicate that device ownership, internet access, digital literacy, language, cultural context, family support, and healthcare team resources determine whether digital interventions are truly accessible (56–58). Without attention to these factors, wearables and AI may widen rather than reduce health disparities.

**Figure 3:**
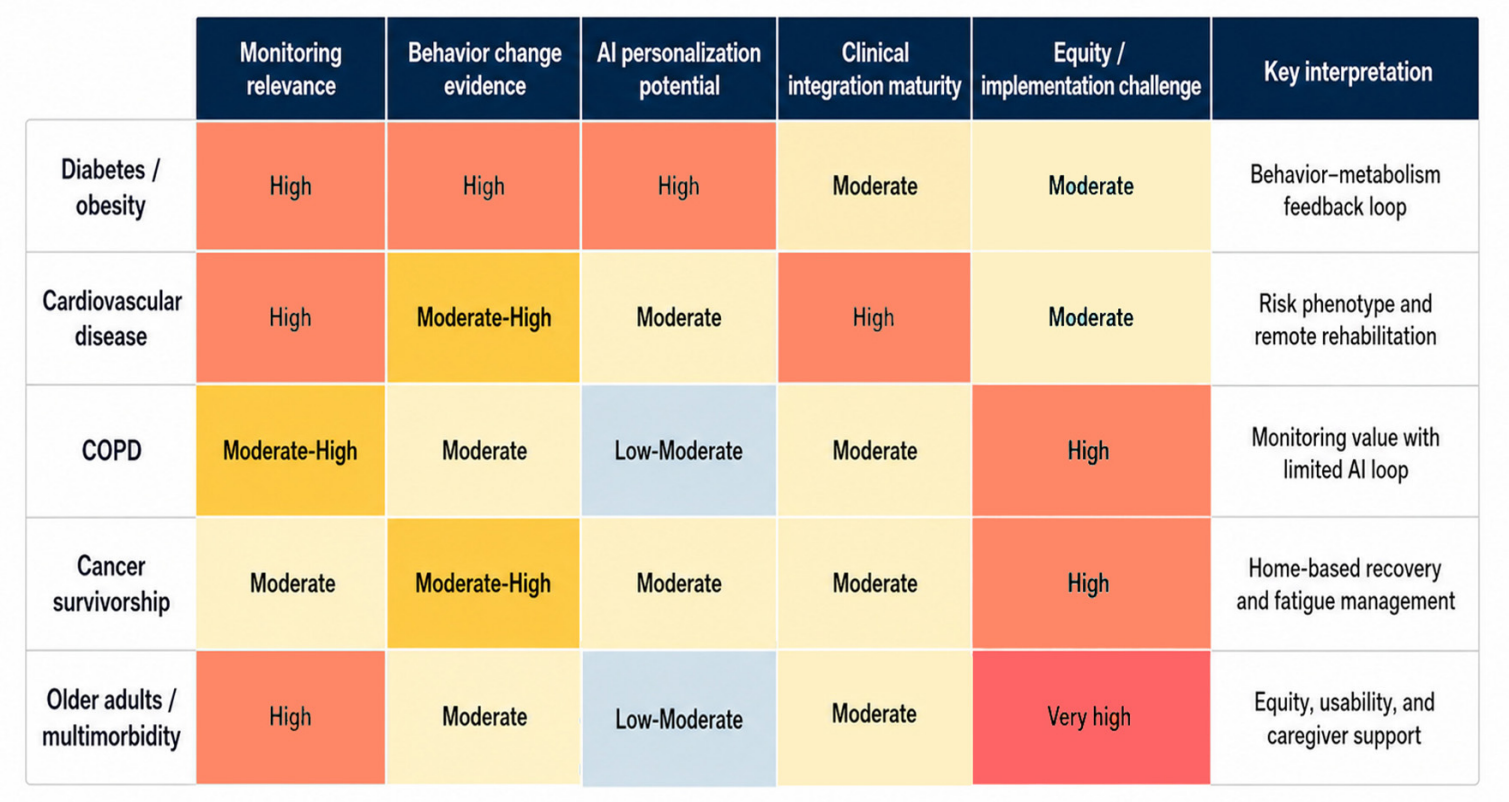
Application landscape of wearable- and AI-enabled physical activity management across chronic disease contexts. The matrix compares major disease contexts in terms of monitoring relevance, behavior change potential, AI personalization potential, clinical integration maturity, and equity or implementation challenge.

Digital interventions for cancer survivors and older adults must also respect illness experience. Cancer survivors may face fatigue, pain, fear of recurrence, body image changes, and social role recovery. Older adults may experience multimorbidity, polypharmacy, fall risk, cognitive decline, sensory impairment, and caregiving dependence. Appropriate targets may therefore involve gradual restoration of daily function, sedentary interruption, self-efficacy, and independent living rather than uniform step thresholds. These populations are not merely special cases; they are core tests of whether digital health can adapt to vulnerability, heterogeneity, and care dependency. Evidence positioning across disease contexts is summarized in Table 2, and Figure 3 proposes a comparative landscape of application contexts.

**Table 2:** Evidence positioning across major chronic disease contexts.

Context	Current evidence positioning	Clinical and research priorities
Diabetes/obesity	Relatively rich evidence on behavioral and selected metabolic outcomes	Emphasize activity tracking, continuous glucose monitoring feedback, and reinforcement learning-enabled personalized intervention.
Cardiovascular diseases	Rapidly growing evidence on hard endpoint associations and remote rehabilitation	Distinguish risk measurement value from causal intervention evidence.
COPD	Evidence supports monitoring and step improvement in pulmonary rehabilitation	Emphasize remote monitoring potential while noting limited AI closed-loop evidence.
Cancer survivorship	Physical activity and sedentary behavior interventions are feasible	Emphasize home-based and remote coaching, fatigue management, and functional recovery.
Older adults/multimorbidity	Behavioral improvement is feasible, but equity and usability are critical	Emphasize digital divide, accessibility, vulnerable populations, and caregiver or community support.

## 5 The added value of AI in wearable-supported management

In chronic disease physical activity management, AI is best discussed through specific functions rather than as a general marker of technological sophistication. Current evidence points to four main roles.

First, AI can support personalized feedback and just-in-time adaptive intervention. Reinforcement learning, user modeling, and just-in-time adaptive interventions can adjust message timing, content, and goals according to history, time, context, and response (12). A scoping review of user models for personalized physical activity interventions identified goal recommendation, activity recommendation, partner recommendation, educational content, motivational content, and intervention timing as personalization modes (13). A recent systematic review of dynamically tailored eHealth interventions for chronic disease further showed that adaptive tailoring has been widely applied to physical activity and healthy-diet support, but also emphasized that tailoring objectives, theoretical foundations, delivery mechanisms, and evaluation designs remain insufficiently standardized (59). Personalization is therefore a testable intervention feature, not evidence of effectiveness by itself.

Second, AI can support exercise prescription and goal setting. Smartwatch-assisted personalized workout recommendation, machine-learning physical activity coaching, and interpretable exercise prescription systems show that multidimensional data can be translated into individualized goals and prescription suggestions (60–62). A systematic review and meta-analysis on machine learning and physical activity-related health outcomes suggests that machine learning may improve intervention matching and behavioral support, but algorithms, inputs, and outcome definitions are heterogeneous (63). The clinical value of these models still depends on whether they improve decisions or outcomes compared with simpler approaches.

Third, AI can support risk prediction and clinical monitoring. AI-enhanced wearable arrhythmia prediction demonstrates that algorithms can identify high-risk events from continuous signals (39). Similar models may support risk stratification, anomaly detection, and reassessment triggers in cardiovascular disease, COPD, and diabetes. But prediction only has clinical value when it triggers defined actions, such as adjusting activity intensity, arranging follow-up, or recommending evaluation.

Fourth, AI can support human–computer interaction and behavioral support. A systematic review of AI chatbots for physical activity, diet, and weight loss included nine studies and 891 participants; five studies suggested potential increases in physical activity, but evidence for diet, weight, and long-term health outcomes was limited and inconsistently reported (64). A meta-analysis of chatbot-based exercise interventions suggests potential in this area (65). A quasi-experimental study of a machine learning-based physical activity chatbot provides additional feasibility and usability evidence, but its evidence level remains lower than long-term randomized trials (66).

Taken together, these functions suggest that AI is most useful when it makes wearable data easier to act on. It can strengthen behavioral feedback and coordinate information flows among patients, devices, algorithms, and clinical teams, but it does not replace medication, clinical judgment, rehabilitation expertise, or social support. Its added value needs to be assessed relative to a comparator, such as no feedback, rule-based reminders, human coaching, or traditional risk scores. Usability or predictive accuracy alone does not establish superiority over simpler alternatives.

Several weaknesses recur across AI studies. External validation is often insufficient, so models trained in a single device ecosystem or population may not generalize. Interpretability also remains limited; patients and clinicians need to understand why reminders, goals, or risk alerts are generated. In addition, clinical action pathways are often incomplete, and prediction has value only when it triggers safe and actionable responses. A scoping review of human factors in AI-driven physical activity solutions further indicates that acceptability, trust, burden, contextual fit, and co-design are frequently underreported, limiting long-term translation (67).

High-level reviews should avoid vague statements such as “intelligent” or “precise.” Instead, they should specify where AI is embedded: data cleaning, activity recognition, risk prediction, goal setting, feedback generation, exercise recommendation, chatbot interaction, or clinical triage. Each role requires different evaluation metrics. Activity recognition requires accuracy and robustness; feedback recommendation requires behavior change and burden assessment; risk prediction requires calibration and decision-curve analysis; chatbots require safety, content quality, empathy, and error-risk monitoring.

## 6 Implementation, governance, and equity: from trial efficacy to health-system use

For public health and health-service research, the key question is how wearable and AI systems can be used in real settings in stable, equitable, interpretable, and sustainable ways.

First, adherence and long-term wearing rates are crucial. Novelty effects may overestimate short-term efficacy. Device forgetting, charging burden, skin discomfort, data fatigue, and feedback fatigue all reduce data completeness. Wearable activity tracker interventions in hospitalized adults suggest that activity promotion is feasible in healthcare institutions, but acute illness, nursing workflow, and discharge transition make adherence more complex than in community interventions (68). Studies should report valid wearing thresholds, missing data handling, dropout reasons, and engagement trajectories. Feasibility and acceptability findings should also be interpreted cautiously because participants who agree to use wearable devices may already have higher digital readiness, motivation, health engagement, or trust in monitoring technologies than the broader chronic disease population.

Second, data quality and comparability are essential. Consumer-device algorithms are updated frequently, and different brands estimate steps, energy expenditure, sleep, and heart rate differently. A living umbrella review on wearable accuracy included 24 systematic reviews, 249 unique validation studies, and 430,465 participants. It found that only a small proportion of consumer wearables had been validated for at least one biological signal, and that validation across all measurable signals remained limited. Heart rate and arrhythmia detection showed better performance, but activity intensity, energy expenditure, and sleep duration had substantial errors (15). Therefore, studies should report device model, algorithm version, wearing position, valid-wear criteria, and missing data procedures. Device capability should also be reported explicitly. Devices without heart-rate monitoring or other physiological sensors may still capture steps or movement volume, but they provide a weaker basis for interpreting activity intensity, exertional safety, recovery patterns, or clinical risk signals. Similarly, higher activity counts from one device should not be automatically interpreted as better or more complete activity capture, because cross-device differences may reflect sensor placement, proprietary algorithms, firmware updates, calibration procedures, or data-cleaning rules rather than true differences in physical activity.

Third, usability and user experience determine adoption. A scoping review of wearable devices and companion mHealth apps included 68 studies, 57 of which used both wearables and connected apps; it identified 29 usability attributes, with satisfaction, ease of use, user experience, perceived usefulness, and effectiveness among the most common, while only seven studies adopted user- or human-centered design paradigms (69). Digital chronic disease management can fail not because sensors are inaccurate, but because interfaces are burdensome, feedback is incomprehensible, training is insufficient, or systems do not fit patients' lives.

Fourth, workflow integration is necessary. Continuous data can increase clinician burden if not translated into defined actions. Implementable systems must specify which indicators trigger alerts, who responds, when exercise prescriptions are adjusted, and when escalation is required. Research on adoption potential in chronic disease management suggests that technology uptake is shaped by perceived benefits, organizational readiness, service process, and support resources (70).

Fifth, equity is central. Older adults, rural residents, low-income groups, minority-language populations, and patients with multimorbidity may face barriers related to device cost, internet access, digital literacy, and caregiving support. Concerns about wearable-device bias and inequity further indicate that unequal device access, population underrepresentation, and algorithmic performance differences may limit the generalizability and fairness of wearable-based health evidence (71). Evidence from urban older adults with chronic diseases suggests that digital access can improve quality of life, but excessive digital use may have negative associations, and social support mediates these relationships. Rural digital health studies similarly show that user factors, provider acceptance, workforce capacity, and payment mechanisms affect implementation (58). Providing devices alone is unlikely to solve equity problems; access, usability, representative data, subgroup evaluation, social support, workforce capacity, and community infrastructure all shape whether these interventions can be used in practice.

Digital health intervention dose is therefore broader than the number of reminders, wearing days, or app logins. It also includes clinical response capacity, patient understanding, family support, organizational resources, and payment mechanisms. A technically sophisticated system will not produce health benefits if clinicians cannot interpret data, payment systems do not support remote management, or patients cannot understand the feedback. Cost and resource allocation also matter. In low-resource settings, the optimal approach may be a low-cost pedometer, text-message feedback, primary care coaching, and simplified algorithms rather than the most advanced device. A pilot randomized trial among hemodialysis patients illustrates that in high-burden, low-activity chronic disease populations, digital physical activity promotion must first demonstrate feasibility, safety, and acceptable patient burden (72).

**Figure 4:**
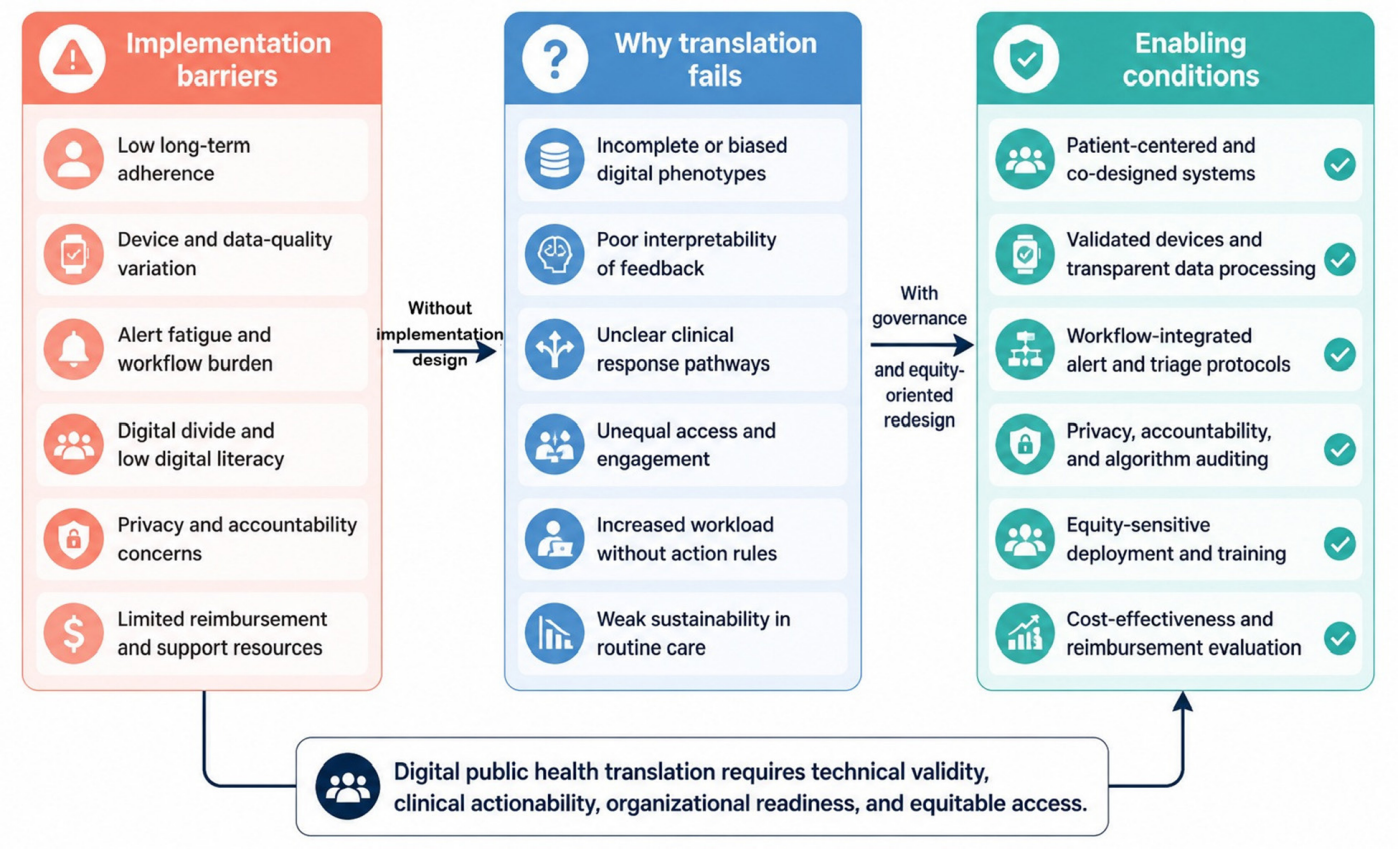
From efficacy to implementation: barriers and enabling conditions for equitable digital public health deployment. The framework connects common barriers, such as adherence decline, data quality variation, workflow burden, privacy concerns, digital divide, and reimbursement limitations, with mechanisms of implementation failure and enabling conditions such as patient-centered design, workflow integration, governance, equity-sensitive deployment, cost-effectiveness evaluation, and training support.

Governance issues include data ownership, secondary commercial use, algorithm updating, clinician responsibility for abnormal data, patient responsibility after ignoring alerts, and regulatory oversight. These are not technical details; they are institutional conditions determining whether digital chronic disease management can become routine care. Figure 4 summarizes implementation barriers and enabling conditions, and Table 3 summarizes key risks and responses.

**Table 3:** Key risks and recommended responses in wearable- and AI-enabled chronic disease physical activity management.

Risk	Manifestation	Recommended response
Evidence overextension	Inferring long-term event reduction from short-term step improvement	Interpret evidence separately across behavioral, functional, hard endpoint, and implementation levels.
Data quality limitation	Device algorithm differences, insufficient wearing time, missing data	Report device model, algorithm version, wearing threshold, and missing data handling.
Algorithmic bias	Lower model performance among vulnerable or underrepresented groups	Conduct external validation and subgroup fairness analysis.
Workflow burden	Continuous data causing alert fatigue and clinical workload	Define trigger thresholds, response responsibilities, and triage protocols.
Digital divide	Older adults, rural residents, and low-income populations facing access barriers	Provide low-threshold devices, training, alternative services, and community support.
Privacy and governance risk	Excessive collection or secondary use of behavioral and physiological data	Clarify data ownership, consent mechanisms, audit processes, and accountability.

## 7 Integrated discussion and future research agenda

### 7.1 Digital closed loops as a new theoretical object

The central argument of this review is that wearable devices and AI should not be understood as isolated patient-facing tools. They should be conceptualized as digital closed-loop infrastructure for chronic disease physical activity management. This framing shifts the research question from “Can a device increase steps?” to “How can continuous behavioral data be translated into individual action, clinical decision-making, and system governance?” Wearables provide high-resolution behavioral and physiological phenotypes; AI generates risk judgments, feedback timing, and personalized recommendations; clinical and community systems determine whether these signals become actionable prescriptions, follow-up, and long-term support.

The first theoretical contribution is an evidence translation hierarchy. Current research often discusses behavior change, risk prediction, and clinical benefit within the same narrative chain, but these outcomes have different evidentiary meanings. Steps, MVPA, and sitting time are behavioral outcomes. Cardiorespiratory fitness, HbA1c, symptoms, and quality of life are functional or intermediate outcomes. MACE, mortality, hospitalization, and exacerbations are hard endpoints. Wearing rate, acceptability, workflow burden, payment, and equity are implementation outcomes. Wearable and AI studies need to establish mediating pathways across these levels rather than using short-term behavior change as a surrogate for long-term clinical benefit.

The second contribution is defining wearable data as algorithmically processed digital phenotypes rather than natural behavioral truths. Device measurement makes lifestyle activity, micro-dosed vigorous activity, sleep rhythms, and all-day movement patterns researchable. Yet consumer device sensors, wearing positions, algorithms, and data-cleaning rules influence estimates, while AI models may amplify data-quality and representativeness problems. Digital phenotypes are valuable not because they replace clinical indicators, but because they provide dynamic, contextual, and feedback-ready representations of behavior and risk.

The third contribution is treating equity, governance, and workflow as internal conditions of digital intervention effectiveness. Traditional efficacy studies often treat digital divide, privacy, reimbursement, clinician burden, and accountability as later-stage implementation issues. In chronic disease physical activity management, these factors directly determine whether the intervention dose is actually delivered. Low digital literacy can prevent wearing and interpretation; under-resourced primary care teams may be unable to process continuous data; alerts without response rules may increase burden; unclear platform-health system responsibilities may limit clinical adoption. Thus, digital effectiveness is generated by patients, families, clinicians, organizations, and governance arrangements, not by devices or algorithms alone.

This definition explains why many digital interventions improve steps in the short term but struggle to demonstrate sustained clinical event reductions. It also explains why improved AI prediction does not automatically generate health benefits. A meaningful digital closed loop must include measurement validity, recommendation appropriateness, clinical actionability, governance accountability, and equitable access.

### 7.2 Future research agenda

Future research should move from proving that digital tools can be useful toward explaining how, for whom, and under what system conditions digital closed loops work.

First, studies should establish mechanisms from digital phenotype to clinical benefit. Trials should prespecify whether wearable feedback improves self-efficacy and goal attainment, whether this increases activity or reduces sedentary time, whether these changes improve cardiorespiratory fitness, glucose variability, symptoms, or functional status, and whether these intermediate changes ultimately influence hospitalization, exacerbation, or cardiovascular events.

Second, future models should address multimorbidity. Real patients often combine metabolic, cardiovascular, respiratory, pain, frailty, and psychological conditions. Future systems should move from single-disease prescriptions to risk-balancing models that determine when to increase activity, when to reduce intensity, and when clinical review is required.

Third, AI evaluation should become explainable, auditable, and actionable. Studies should report calibration, external validation, subgroup performance, error consequences, human review mechanisms, and clinical action rates. In exercise prescription, algorithms must be linked to contraindications, symptoms, hypoglycemia risk, arrhythmia, frailty, and patient burden.

In addition, the added value of AI should be tested against explicit comparators, including no feedback, rule-based reminders, traditional risk scores, and human coaching.

Fourth, equity should be designed into trials from the beginning. Age, sex, rurality, income, education, language, digital literacy, disability, and comorbidity burden should be considered at recruitment, intervention design, and analysis stages. Equity is not a post hoc subgroup exercise; it shapes device selection, training, feedback language, family support, alternative services, and cost coverage.

Fifth, the field should compare service models rather than products. Health systems need to know which combinations of device, algorithm, coaching, clinical follow-up, and payment are effective and sustainable. Comparisons should include low-cost pedometers with text feedback, smartwatches with remote coaching, AI triage with human review, and community-supported versus hospital-led delivery models.

Sixth, regulation, payment, and data governance should be treated as mainstream evidence domains. Digital physical activity management depends on continuous behavioral and physiological data. Without clear accountability and payment mechanisms, clinicians will not reliably respond to data. Without auditing and safety monitoring, AI recommendations will not earn trust. Without data governance, patients may not authorize long-term use.

## 8 Conclusion

Wearable devices and AI do not replace clinical services. Their core value lies in providing continuous digital phenotypes, personalized behavioral feedback, and remote coordination infrastructure for chronic disease physical activity management. As digital health solutions, they are most relevant when they help health systems extend monitoring, coaching, rehabilitation, and follow-up beyond episodic clinical encounters. The most robust evidence supports improvements in steps, MVPA, sedentary behavior, and self-monitoring. Evidence for cardiorespiratory fitness, HbA1c, quality of life, and readmission is promising but heterogeneous. Evidence for MACE, mortality, long-term complications, and system-level cost-effectiveness requires stronger causal validation.

The central implication is that digital physical activity management should be evaluated as a closed-loop service process rather than as a collection of devices or apps. Short-term behavior change is more likely to translate into long-term health benefit when digital phenotypes are reliable, AI recommendations are interpretable, clinical actions are executable, implementation costs are sustainable, and vulnerable populations have equitable access. Because this is a problem-oriented narrative synthesis rather than a systematic review or meta-analysis, and because it is based mainly on English-language and accessible published evidence, the conclusions should be interpreted cautiously, especially in relation to non-English implementation contexts, unpublished evidence, and rapidly evolving AI applications. The non-systematic source selection also creates potential selection and citation bias, even though the revised search log and SANRA-informed reporting were used to make the evidence-identification process more transparent. Future research should focus less on adding technological functions and more on validating whether these digital health pathways can improve health safely, fairly, and sustainably in real chronic disease populations and real health systems.
