# Supplementary material for: Digital health solutions for chronic disease physical activity management: wearable devices, artificial intelligence, and public health implementation
Source: Front Public Health. 2026 Aug 4;14:1888001. doi: 10.3389/fpubh.2026.1888001 (PMC13481783; doi:10.3389/fpubh.2026.1888001)
Supplement: Supplementary file 1 [file Data_Sheet_1.pdf]

Supplementary Table S1. Search strategy and source-selection log

Search theme	Source	Search string / approach	Search date	Records
Wearables, chronic disease, and physical activity	Web of Science Core Collection	TS=((”wearable device*” OR smartwatch* OR ”activity tracker*” OR ”fitness tracker*” OR accelerometer*) AND (”physical activity” OR exercise OR ”sedentary behavior” OR rehabilitation OR ”self-management”) AND (”chronic disease” OR diabetes OR obesity OR ”cardiovascular disease” OR COPD OR cancer OR ”older adults” OR multimorbidity))	25 Mar 2026	68
Metabolic disease, diabetes, obesity, and activity tracking	Web of Science Core Collection	TS=((”wearable device*” OR ”activity tracker*” OR ”continuous glucose monitoring” OR CGM OR mHealth) AND (”type 2 diabetes” OR obesity OR cardiometabolic) AND (”physical activity” OR exercise OR sedentary OR ”self-management”))	27 Mar 2026	47
Cardiovascular disease, cardiac rehabilitation, and wearable monitoring	Web of Science Core Collection	TS=((wearable* OR smartwatch* OR accelerometer* OR ”activity tracker*”) AND (”cardiac rehabilitation” OR ”cardiovascular disease” OR ”heart failure” OR ”coronary artery disease” OR MACE) AND (”physical activity” OR exercise OR rehabilitation OR monitoring))	30 Mar 2026	45
COPD, pulmonary rehabilitation, and remote monitoring	Google Scholar	Phrase searches combining ”COPD”, ”pulmonary rehabilitation”, ”wearable”, ”activity tracker”, ”physical activity”, ”remote monitoring”, ”digital health”, and ”randomized trial” or ”meta-analysis”; relevance-ranked results were screened.	02 Apr 2026	39
Cancer survivorship, older adults, multimorbidity, and equity	Google Scholar	Phrase searches combining ”cancer survivors”, ”older adults”, ”multimorbidity”, ”wearable activity tracker”, ”physical activity”, ”mHealth”, ”digital divide”, ”equity”, ”rural”, and ”chronic disease”; relevance-ranked results were screened.	04 Apr 2026	44
Artificial intelligence, adaptive intervention, and behavioral support	Google Scholar	Phrase searches combining ”artificial intelligence”, ”machine learning”, ”reinforcement learning”, ”chatbot”, ”just-in-time adaptive intervention”, ”personalized feedback”, ”physical activity”, and ”chronic disease”; relevance-ranked results were screened.	06–08 Apr 2026	54
Implementation, governance, device accuracy, and human factors	Web of Science Core Collection / Google Scholar	Topic searches combining ”wearable accuracy”, ”device validation”, ”data governance”, ”workflow integration”, ”implementation”, ”human factors”, ”AI”, ”equity”, ”physical activity”, and ”chronic disease management”.	10–12 Apr 2026	37
Backward citation tracking and citation-status verification	Reference lists and publisher pages	Reference lists of key umbrella reviews, systematic reviews, meta-analyses, professional statements, and implementation-oriented papers were checked; final publication status was verified for 2026 references before submission.	13 Apr–18 May 2026	22

Source-selection stage	Number of records
Records/items identified from thematic Web of Science, Google Scholar, and citation-tracking searches	356
Records/items remaining after duplicate removal and preliminary relevance consolidation	248
Records/items screened by title/abstract and topic relevance	248
Full texts assessed for relevance to the narrative synthesis	112
Core sources retained and cited in the manuscript	71

Note: Searches were conducted before the original submission date of 22 May 2026. The main database searches were performed across topic-specific searches in Web of Science Core Collection and Google Scholar from 25 March to 12 April 2026, followed by backward citation tracking, eligibility checking, and final citation-status verification through 18 May 2026. This log was used to make the source-identification process transparent for a SANRA-informed narrative review. It is not intended to present the review as a PRISMA-style systematic review or meta-analysis.
